# Short Report: Autism in Popular Media: A Google Trends Analysis of Public Interest

**DOI:** 10.1177/13623613261489305

**Published:** 2026-09-24

**Authors:** Kevin Rebecchi, Anders Nordahl-Hansen

**Affiliations:** 1 Østfold University College, Halden, Norway

**Keywords:** autism, Asperger, google trends, popular media, public interest, media representation, online search behaviour

## Abstract

Public interest in autism-related topics can be tracked through online search behaviour, yet little is known about how popular media portrayals structure this interest over time. This study analysed Google Trends data for *autism* and *Asperger* between 2004 and 2025, focusing on popular media-related results within the categories *Movies* and *TV & Video*. Results showed a progressive increase in search interest for autism and a decline for Asperger in both categories, alongside a marked concentration of attention around a small number of highly recurrent media references. Male-coded characters dominated the sample, although several recent female portrayals generated substantial interest. These findings highlight the role of popular media in shaping the terms, figures and representations through which autism becomes visible online.

**Lay Abstract:**

This study looked at how people search online for information about autism and Asperger’s, and how popular movies and TV shows influence that interest. By analysing Google Trends data from 2004 to 2025, we found that searches for autism have steadily increased, while searches for Asperger have declined. A small number of media portrayals accounted for most of the search activity, showing that certain portrayals strongly shape public curiosity. Most of these characters were male, although interest in female autistic characters has grown in recent years. Overall, the findings suggest that popular media play an important role in how the public learns about and understands autism, influencing which terms, stories and representations become most visible online.

## Introduction

Popular media remain a major source through which the public encounters and interprets autism. Portrayals in popular media can increase visibility, raise awareness and acceptance, but may also reinforce narrow or stereotyped understandings of autism, particularly through repeated associations with savant skills or socially eccentric male characters (Jones et al., 2023; Nordahl-Hansen et al., 2018). Recent reviews further suggest that autism representation continues to shape public attitudes, knowledge and stigma, even when portrayals appear more visible or ostensibly positive (Mittmann et al., 2024).

In parallel, infodemiological studies have shown that public interest in autism-related topics can be meaningfully tracked through Google Trends. This tool provides relative search volume indices (0–100) normalized to peak search volume, allowing researchers to track and compare public interest in health-related terms across time and geography. Prior work has examined seasonal peaks in autism searches, especially during awareness campaigns (Lang-Illievich et al., 2023), long-term changes in web searches concerning autism causes and treatments (Saposnik & Huber, 2020), and event-driven fluctuations in searches for *autism* and *Asperger syndrome* associated with media coverage and public figures (Skafle et al., 2021; Tárraga-Mínguez et al., 2020). More recently, country-specific analyses have confirmed the sustained growth of online interest in autism over time (Akdağ et al., 2025). At the same time, a growing body of research has documented how autism is discussed on digital platforms such as TikTok and Instagram, where highly viewed content may combine awareness-raising, self-disclosure, stereotyping and misinformation (Aragon-Guevara et al., 2023; Brown et al., 2024; Carollo et al., 2026; Ononuju & Ujari, 2025; Steiner-Hofbauer et al., 2025).

However, despite this expanding literature on both media representation and digital search behaviour, little is known about how popular media may structure public interest in autism-related terms over time. To our knowledge, no study has specifically examined Google Trends related to *autism* and *Asperger* from the perspective of the most salient popular media portrayals associated with these search dynamics. The present study will thus address this gap.

## Methods

Google Trends Explore was used to search the terms *autism* and *Asperger* as free-text search terms, filtered by the *Movies* and *TV & Video* categories, at the worldwide level, year by year from 2004 to 2025. For each search, the ‘Related Topics’ and ‘Related Queries’ (Top) sections were reviewed, and the displayed interest score (0–100) for each result was recorded manually by the first author and validated through discussion with the second author.

Items were included when results referred to an identifiable fictional media title or character for whom autism or autistic traits were central to the character or narrative, even when no formal diagnosis is stated within the story. Documentary content was also included when it featured autistic individuals or autism as its primary subject, given that documentaries constitute a significant mode through which autism is represented and encountered in popular media. Results referring to real individuals were excluded (e.g., Tim Burton), as were cases in which autism was not sufficiently central. For instance, in *Sitaare Zameen Par*, the main character works with neurodivergent children, but no individual character’s autism is foregrounded in the narrative. Where multiple labels referred to the same item (e.g., ‘Sheldon’, ‘Sheldon Cooper’, ‘Big Bang Theory’), results were harmonized under a single label (e.g., ‘Leonardo DiCaprio’ was recorded under ‘What’s Eating Gilbert Grape’). Where the Google Trends result included a character name alongside the title, both are reported in Table 1; where only the title appeared in the results, no specific character is identified.

**Table 1.** Descriptive Summary of Media Titles and Interest Scores.

Title	Released year	Genre	Gender	Frequency	Mean interest score	Total interest score
*The Big Bang Theory* (Sheldon Cooper)	2007	TV Series	Male	32	61.78	1,977
*The Curious Incident of the Dog in the Night-Time* (Mark Haddon)	2003/2012	Book (2003), Play (2012)	Male	32	48.66	1,557
*Sesame Street* (Julia)	2017	Animation / Cartoon	Female	11	88.64	975
*Autism: The Musical*	2007	Documentary	Both	13	38.15	496
*My Name Is Khan*	2010	Movie	Male	24	18.54	445
*Adam*	2009	Movie	Male	13	26	338
*The Good Doctor*	2017	TV Series	Male	18	13.83	249
*Forrest Gump*	1994	Movie	Male	19	10.47	199
*True Life* (Episode: “I have autism”)	2007	TV Series	Male	6	30	180
*Extreme Love: Autism* (Louis Theroux)	2012	Documentary	Male	4	43.75	175
*Somebody*	2022	TV Series	Male	6	21.5	129
*Inside Our Minds* (Chris Packham)	2023	Documentary	Both	1	78	78
*Parenthood*	2010	TV Series	Male	5	15	75
*Temple Grandin*	2010	Movie	Both	5	13.8	69
*EastEnders*	1985	TV Series	Both	3	22.67	68
*South Park*	2011	Animation / Cartoon	Male	4	14.75	59
*Refrigerator Mothers*	2002	Documentary	Both	2	25	50
*Autism in Love*	2015	Documentary	Both	2	18	36
*Planeta Asperger*	2014	Documentary	Both	4	8.5	34
*Young Sheldon*	2017	TV Series	Male	1	28	28
*Move to Heaven*	2021	TV Series	Male	4	6.75	27
*La Rosa de Guadalupe*	2008	TV Series	Male	3	8.33	25
*Extraordinary Attorney Woo*	2022	TV Series	Female	1	23	23
*Ella Schon* (Annette Frier)	2018	Movie	Female	4	5.5	22
*The A Word*	2016	TV Series	Male	1	21	21
*Atypical*	2017	TV Series	Male	1	20	20
*Malhação: Viva a Diferença* (Benê)	2017	TV Series	Female	2	10	20
*Power Rangers* (Billy Cranston, Blue)	1993	Movie	Male	1	19	19
*Paul Blart: Mall Cop*	2009	Movie	Male	2	9	18
*What’s Eating Gilbert Grape* (Leonardo DiCaprio)	1993	Movie	Male	2	8	16
*Float* (Pixar)	2019	Animation / Cartoon	Male	1.5	9	13.5
*A Is for Autism*	1992	Documentary	Both	1	13	13
*Mary and Max*	2009	Animation / Cartoon	Male	2	6.5	13
*Music* (Sia)	2020	Movie	Female	2	6.5	13
*Loop* (Pixar)	2020	Animation / Cartoon	Female	1.5	8.33	12.5
*Autism × 6*	2010	Documentary	Both	1	12	12
*Finding Dory*	2016	Animation / Cartoon	Female	1	12	12
*Bones*	2005	TV Series	Female	2	5.5	11
*The Rehearsal*	2022	TV Series	Male	1	9	9
*I Am Sam*	2001	Movie	Male	2	4	8
*Dr House*	2004	TV Series	Male	1	6	6
*Austin Powers*	1997	Movie	Male	1	5	5
*La mujer perfecta* (The Perfect Woman)	2010	TV Series	Female	1	5	5
*The Predator*	2018	Movie	Male	1	5	5
*Lilo & Stitch*	2002	Animation / Cartoon	Female	1	5	5
*Life, Animated*	2016	Documentary	Male	1	4	4
*Rain Man*	1988	Movie	Male	1	3	3

Cross-media properties were included when their adaptation generated Google Trends results classified under the Movies or TV & Video categories and autism was central to the character’s portrayal; *The Curious Incident of the Dog in the Night-Time* was retained on this basis. Each included media item was coded by the first author as involving male, female, or both male and female characters, based on the sex of the character(s) explicitly associated with autism or autistic traits in the narrative.

The Interest Total Score was calculated by summing all recorded interest scores across annual appearances; the Interest Mean Score was obtained by dividing this total by the frequency of appearance. In addition to identifying the media items most frequently associated with these terms, we compared overall search interest over time and examined geographic variation across world regions.

## Results

The final sample (Table 1) comprised 48 results, including TV series (*n* = 17), movies (*n* = 13), documentaries (*n* = 9), animation/cartoons (*n* = 7) and one book/play adaptation (*n* = 1).

Media items were coded as involving male characters (*n* = 28), female characters (*n* = 10), or both male and female characters (*n* = 9). The frequency of appearance of each media item in the Trends-related corpus ranged from 1 to 32. Seventeen media items appeared only once, whereas the highest observed frequency was 32, recorded for both *The Big Bang Theory* (Sheldon Cooper) and *The Curious Incident of the Dog in the Night-Time* (Mark Haddon).

The Interest Total Score ranged from 3 for *Rain Man* to 1,977 for *The Big Bang Theory* (Sheldon Cooper). The Interest Mean Score ranged from 3 for *Rain Man* to 88.6 for *Sesame Street* (Julia). Data also indicate a progressive increase in public interest in *autism* across both Movies and TV & Video from 2004 to 2025 (Figure 1), particularly from the late 2010s onward. In contrast, interest in *Asperger* decreased in both categories over the same period, suggesting a gradual shift in the terminology used in media-related search behaviour.

**Figure 1.**
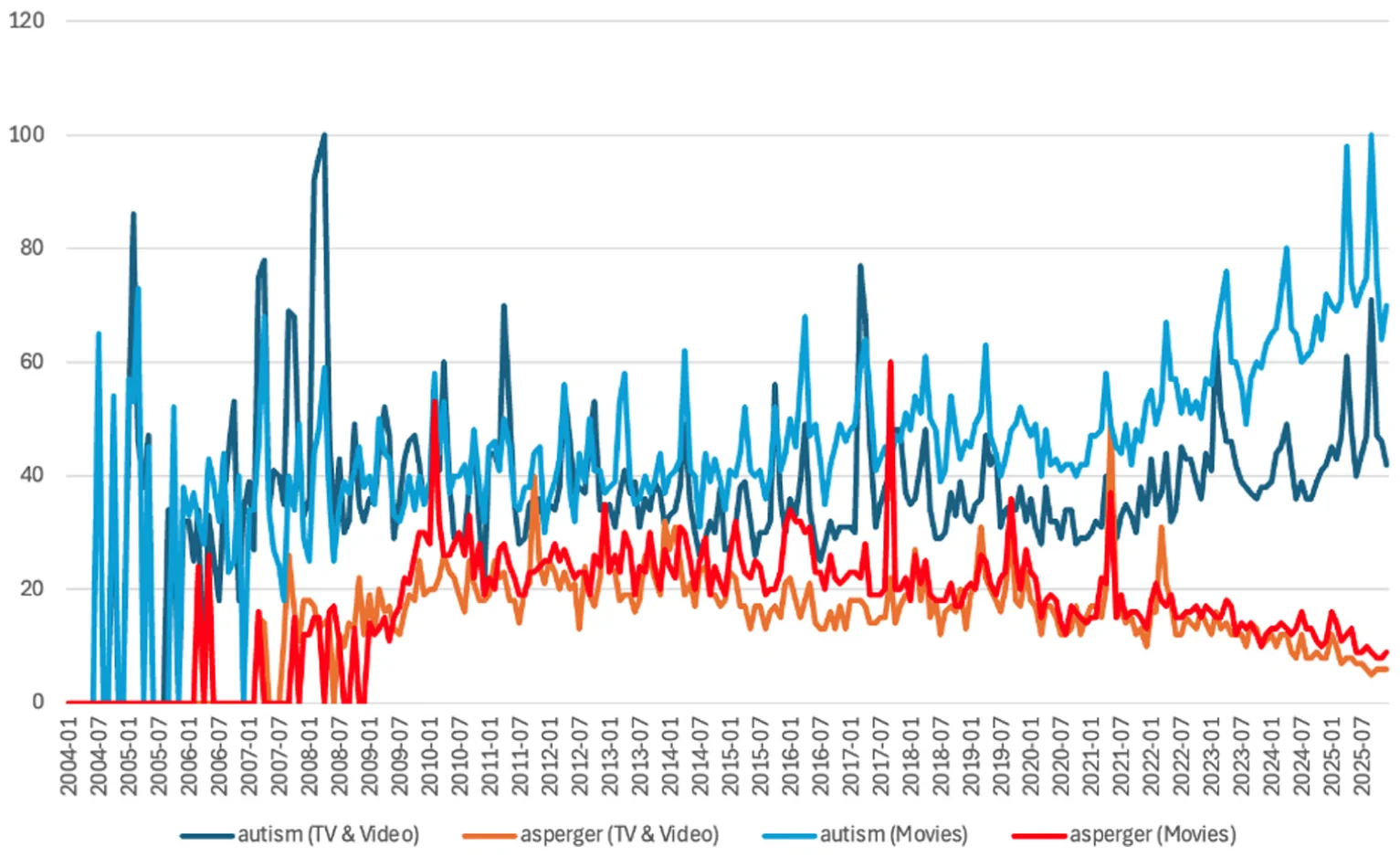
‘Autism’ and ‘Asperger’ research interest over time (2004–2025).

The geographic distribution (Figure 2) of search interest (similar in both categories) also differed between the two terms. Search interest in *Asperger* appeared to be more concentrated in Latin-language countries, particularly in parts of Southern Europe and Latin America, whereas *autism* showed a broader distribution across the rest of the world.

**Figure 2.**
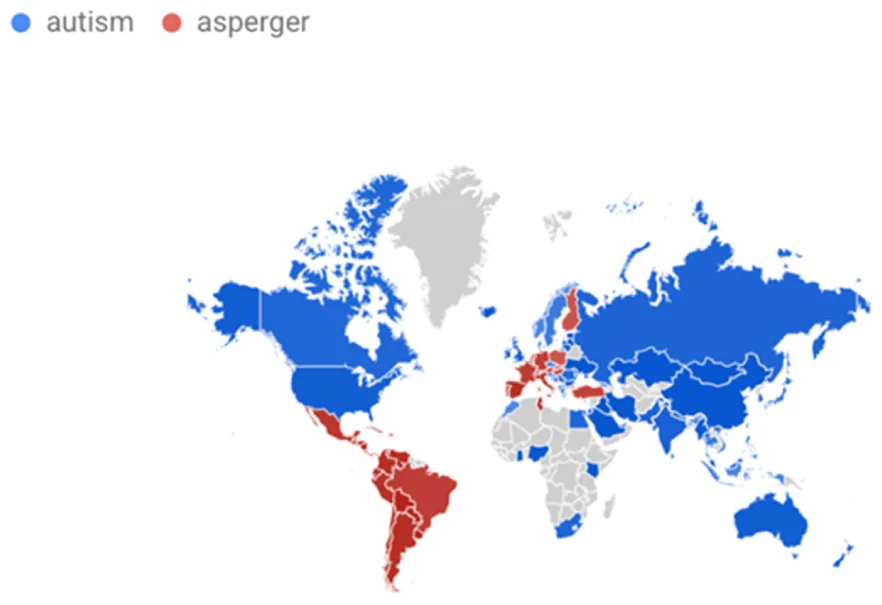
‘Autism’ and ‘Asperger’ geographic distribution of relative search interest over time (2004–2025).

## Discussion

A first notable result is the sex imbalance in the media products identified. Male characters clearly dominated the sample, whereas female characters remained comparatively underrepresented (ratio 1:3). This pattern is consistent with long-standing observations in both clinical and media research (Dean, Nordahl-Hansen, 2022); however, the present data also suggest a gradual change over roughly the last decade, with increased visibility of female-coded characters such as Julia in *Sesame Street*, Woo Young-woo in *Extraordinary Attorney Woo* and other more recent portrayals highlighted in research (Rebecchi, 2025; Tharian et al., 2019).

A second important finding concerns the disparity between canonical status in academic or clinical discourse and apparent visibility in public search behaviour. *Rain Man*, for example, is often treated as the iconic autism reference (Ikalyuk & Kuzmyn, 2015), yet in the present dataset, it was among the least recurrent and least popular items. Conversely, titles such as *The Big Bang Theory*, *The Curious Incident of the Dog in the Night-Time* and *Sesame Street* generated far stronger cumulative search associations. This suggests that the classic reference points do not necessarily coincide with the media objects that structure contemporary online curiosity and interest.

Moreover, some characters frequently discussed in relation to autism (Nordahl-Hansen, 2017) or autistic traits (*The Bridge*, *Community*, *Mozart and the Whale*, *Bones*, *Dr. House* and *Atypical*) appeared rarely or not at all. Likewise, *Music* by Sia, despite intense public debate and criticism, in particular by autistic people (Orm et al., 2023), appeared only twice. Although documentaries differ generically from fictional portrayals, their inclusion reflects the broader aim of capturing the full range of popular media formats through which autism becomes visible in public search behaviour. Google Trends captures one specific mode of public engagement, and this likely underrepresents media discussions and visibility that unfold primarily on other platforms (TikTok, Reddit and IMDb, for instance). In addition, the analysis was limited to the terms *autism* and *Asperger*; the Google Trends interface does not support Boolean or wildcard queries, precluding the inclusion of related terms such as autism spectrum disorder (ASD). Future studies may consider extending the search to additional terminology.

The temporal contrast between *autism* and *Asperger* is also clear. Across both categories *Movies* and *TV & Video*, interest in *autism* increased progressively from 2004 to 2025, especially from the late 2010s onward, whereas interest in *Asperger* declined. This pattern likely reflects a broader terminological and cultural shift. Since Asperger’s syndrome was removed as a separate diagnostic category in the *Diagnostic and Statistical Manual of Mental Disorders* (*DSM*-5) in 2013 and folded into ASD, the term has gradually lost institutional centrality even if it has remained visible in public discourse. In addition, controversies surrounding Hans Asperger and renewed critical discussion of the historical and ethical implications of the label (see Rebecchi, 2024, for a discussion about this topic) may also have contributed to its declining use. However, the geographic differences between the two terms may suggest that the replacement of *Asperger* by *autism* has not occurred uniformly across linguistic and cultural contexts (both in research and popular media). The geographic distribution of search interest should nonetheless be interpreted with caution. While Asperger functions as a proper name and circulates across languages, the term *autism* is an English-language word; speakers of other languages are more likely to search for equivalent terms (e.g., autismo, autisme, Autismus). The apparent concentration of autism searches in English-speaking regions may, therefore, partly reflect linguistic factors, and cross-regional comparisons between the two terms should be read accordingly.

Finally, one particularly intriguing case is Tim Burton, which was excluded for not being a fictional character or media product in the strict sense. Searches involving Tim Burton may reflect public interest in reported statements or speculation concerning his own possible autism; however, they may also reflect attempts to interpret autism through his films or through universe and characters perceived as atypical or outsider figures in Burton’s cinematic universe (Edward Scissorhands, Sweeney Todd, Alice, Miss Peregrine’s Home).

These findings also carry practical implications. Popular media portrayals of autism feed into the representations that teachers, journalists and communication professionals encounter and reproduce. University curricula in teacher training and journalism could benefit from integrating media literacy components that explicitly address how autism is framed in fictional content, which characters dominate public attention and how these portrayals diverge from clinical and experiential realities. This is particularly relevant given that even reference sources such as Wikipedia reproduce comparable patterns across languages (framing autism primarily as a childhood, biomedical condition and marginalizing neurodiversity and adult perspectives, see Rebecchi et al., 2026) suggesting that the issue is not confined to entertainment media but extends to the broader online information ecosystem that shapes professional knowledge.

Public search patterns indicate that popular media play a substantial role in shaping how people engage with autism online, with some fictional characters driving far more interest than others. The results also show a clear gender imbalance, though recent portrayals suggest a slow increase in female representation. Several characters widely discussed in academic or fan-communities generated little search activity, highlighting a gap between expert discourse and public curiosity. Over time, searches for ‘autism’ have risen steadily, while interest in ‘Asperger’ has declined, reflecting both diagnostic changes and shifting cultural attitudes. Geographic differences further suggest that this transition is uneven across regions.

## Data Availability

Researchers may request access to the data by contacting the corresponding author. The corresponding author obliges to respond to any such request.*
